# Coexistence of rectal adenocarcinoma and rectal and lymph node tuberculosis: a case report with a limited narrative review

**DOI:** 10.3389/fonc.2026.1819975

**Published:** 2026-06-11

**Authors:** Sanbao Shi, Da Li, Peng Xu, Cheng Zhang

**Affiliations:** Department of General Surgery, General Hospital of Northern Theater Command, Shenyang, Liaoning, China

**Keywords:** case report, literature review, lymph node tuberculosis, rectal cancer, rectal tuberculosis

## Abstract

**Background:**

Rectal cancer concurrent with regional tuberculosis is a rare clinical entity. Due to its similar clinical symptoms and imaging features, it is frequently misdiagnosed, which profoundly complicates patient management.

**Case presentation:**

A 72-year-old man with a history of pulmonary tuberculosis presented with three months of intermittent hematochezia and tenesmus. Because the patient self-reported the tuberculosis as cured and initial imaging showed no active pulmonary or evident extrapulmonary lesions, preoperative molecular tuberculosis screening was not initiated. Preoperative imaging and biopsy confirmed rectal adenocarcinoma, alongside clinically suspected (but not histologically proven) pulmonary metastases. After neoadjuvant chemoradiotherapy, further chemotherapy was halted due to severe myelosuppression. A Hartmann procedure was performed after stable disease was confirmed. Postoperative pathology showed rectal adenocarcinoma with no lymph node metastasis (0/24) but unexpectedly revealed granulomatous inflammation in lymph nodes and rectal tissue. TB DNA PCR was positive, providing molecular evidence supporting concurrent tuberculosis. The patient developed postoperative anal discharge and a clinically suspected tuberculosis-related presacral infectious process, which improved after local irrigation and systemic anti-tuberculosis therapy. At one year, there was no tumor recurrence or new tuberculosis.

**Conclusion:**

In patients with a history of pulmonary tuberculosis who present with rectal cancer, the possibility of concurrent rectal and lymph node tuberculosis should be carefully considered. This case highlights the significant diagnostic overlap between these conditions, the high risk of delayed tuberculosis recognition, and its critical impact on postoperative management. Maintaining a high index of suspicion and utilizing comprehensive pathological and molecular evaluations are essential for accurate differential diagnosis and optimized patient care.

## Introduction

Rectal cancer is one of the most common malignancies of the gastrointestinal tract, with incidence rates steadily rising worldwide. The standard treatment typically involves a combination of surgery, chemotherapy, and radiotherapy, often tailored according to tumor stage and patient condition (1). Early diagnosis and appropriate multidisciplinary management are critical for improving outcomes.

Extrapulmonary tuberculosis (TB), particularly intestinal tuberculosis, has shown a rising trend in recent years despite an overall decline in pulmonary TB incidence (2). Intestinal TB most commonly affects the ileocecal region but can involve any segment of the gastrointestinal tract, including the rectum. Its clinical presentation is often nonspecific and may mimic various diseases, including malignancies and inflammatory bowel disease, making accurate diagnosis challenging (3).

Differential diagnosis between rectal cancer and intestinal tuberculosis is particularly difficult due to overlapping features. Both conditions can present with similar clinical symptoms such as hematochezia and tenesmus. Imaging studies may reveal intestinal wall thickening and lymphadenopathy in both diseases (4). Endoscopic findings are also nonspecific, with both conditions capable of causing mucosal irregularities, ulceration, or mass-like lesions. As a result, relying solely on clinical, imaging, or endoscopic assessment is often insufficient for accurate differentiation (5). As highlighted in recent literature, gastrointestinal tuberculosis lacks a single, highly accurate diagnostic algorithm; thus, preventing delayed diagnosis and subsequent complications requires a high index of suspicion and the rigorous integration of clinical, radiological, endoscopic, histopathological, and microbiological findings (6).

Here, we report a rare case of rectal cancer with concurrent rectal and lymph node tuberculosis. While it is well-documented that intestinal tuberculosis can mimic malignancy (7), this report goes beyond generic diagnostic overlap to explicitly define the complex management problems this coexistence creates. Specifically, this case illustrates how concurrent tuberculosis can actively alter tumor staging impressions (e.g., mimicking peritoneal or nodal metastases), significantly influence the sequencing of multidisciplinary treatments, and severely complicate the interpretation of postoperative events. By sharing these insights, our report aims to raise clinical awareness of this rare entity and provide a stronger, literature-based framework for its diagnosis and management.

## Case description

A 72-year-old male with a 3-year history of hypertension and a 5-year history of pulmonary tuberculosis presented with intermittent hematochezia and tenesmus for over three months (Month 0). In accordance with CARE guidelines, a comprehensive patient history was obtained. The patient had a significant smoking history of approximately 50 years, consuming about 30 cigarettes per day. Since the onset of his current illness, he did not experience any obvious weight loss or other constitutional symptoms (such as fever or night sweats). His hypertension was well-controlled with a daily combination tablet of valsartan and amlodipine (1 tablet/day), and he was not taking any other medications or immunosuppressants at the time of presentation. Regarding his tuberculosis history, the patient self-reported that his pulmonary tuberculosis from 5 years prior had been completely cured; however, the specific anti-tuberculosis regimen he previously received was unknown. He had not experienced any recent TB-related respiratory or systemic symptoms. Furthermore, his family and psychosocial history were unremarkable, with no family history of colorectal cancer and no recent contact with active tuberculosis patients at home. Upon initial physical examination, the patient’s vital signs were stable, and he was afebrile. His general appearance and performance status were fair, consistent with his age and overall condition. There was no obvious evidence of severe malnutrition or cachexia. Notably, there were no systemic signs of active infection, and abdominal examination was unremarkable with no palpable masses or tenderness. Anorectal examination revealed no perianal fistulas, abscesses, or other localized signs of infection. Digital rectal examination (DRE) was negative for a palpable tumor or stricture. Prior to admission, a pelvic contrast-enhanced MRI performed at an outside institution suggested locally advanced rectal cancer (cT4N+). Subsequent abdominal contrast-enhanced CT at our hospital confirmed a rectal mass measuring 4.5 cm from the anal verge, circumferentially involving the intestinal lumen, with blurred perirectal fat planes and enlarged paraintestinal lymph nodes, consistent with cT4bN2 locally advanced rectal cancer. Chest CT showed a solitary nodule in the left lung. This pulmonary lesion was carefully evaluated by a multidisciplinary team (MDT), including thoracic surgeons and radiologists. Based on the imaging characteristics, they concluded that while there were benign, old pulmonary lesions consistent with the patient’s history of tuberculosis, the solitary nodule was strongly suspected to be a pulmonary metastasis. No imaging signs of active pulmonary tuberculosis were observed. Although lacking initial tissue confirmation, this strong clinical suspicion of metastasis guided the subsequent decision for a palliative surgical approach. Although the patient had a 5-year history of pulmonary tuberculosis, he reported the disease had been fully cured. Preoperative pulmonary imaging did not show any signs of active tuberculosis, and the potential features of extrapulmonary tuberculosis in the intestine were entirely masked by the locally advanced rectal cancer. Consequently, routine molecular testing (such as PCR) or more rigorous tuberculosis screening was not initiated prior to surgery. Colonoscopy and pathology confirmed rectal adenocarcinoma, with additional scattered areas of pale red mucosa in the distal rectal wall. However, these pale areas were not separately biopsied at the time, as they were visually interpreted by the endoscopist as peritumoral inflammatory changes or routine mucosal irritation secondary to bowel preparation. Tumor markers were mildly elevated: CEA 4.42 mg/L (reference: 0–5 mg/L), CA199 19.85 U/mL (reference: 0–37 U/mL), and CA24-2 8.19 IU/mL. After multidisciplinary consultation, the patient was diagnosed with locally advanced rectal cancer with suspected pulmonary metastasis, and neoadjuvant therapy followed by surgery was recommended.

**Figure 1:**
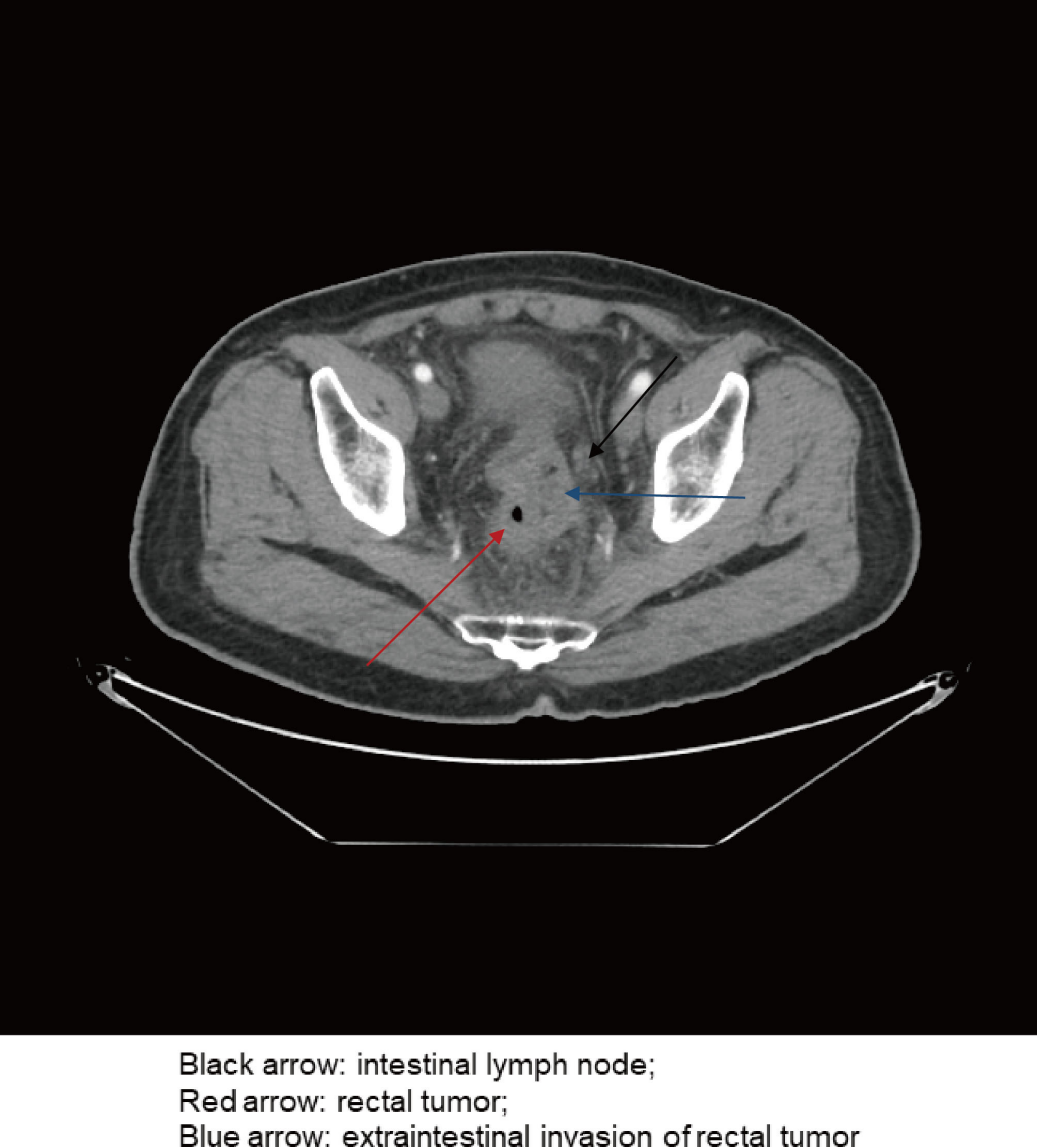
Post-neoadjuvant pelvic CT. The red arrow indicates the rectal tumor mass. The blue arrow points to suspected extramural invasion involving the perirectal fat. The black arrow shows an enlarged pararectal lymph node, suggesting persistent nodal disease (N2) despite neoadjuvant therapy.

**Figure 2:**
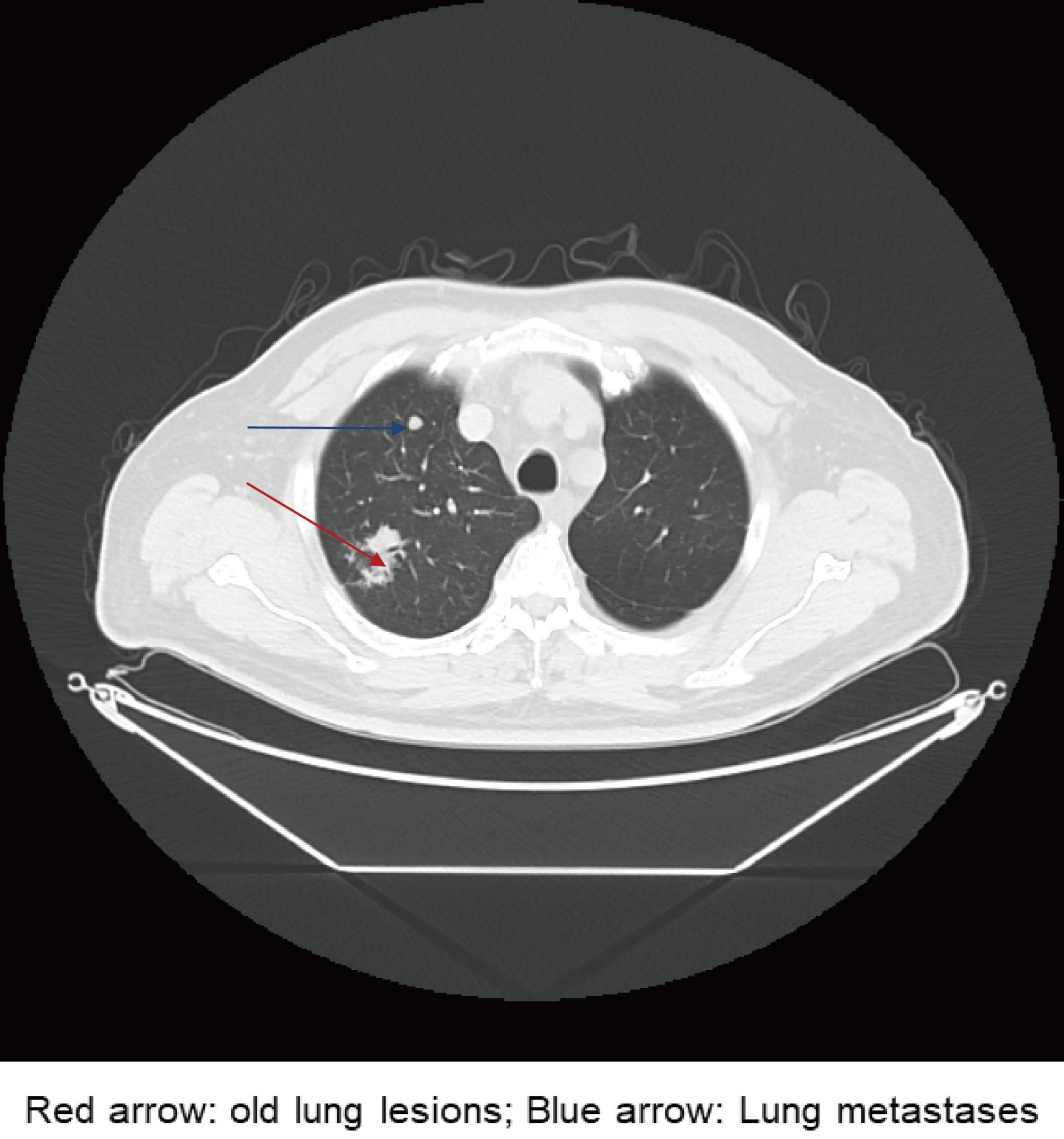
Post-neoadjuvant Chest CT. The blue arrow indicates a solitary nodule in the left lung, which was clinically suspected to be a pulmonary metastasis. The red arrow points to a benign, old pulmonary lesion (calcification/fibrosis) consistent with the patient's history of tuberculosis.

**Figure 3:**
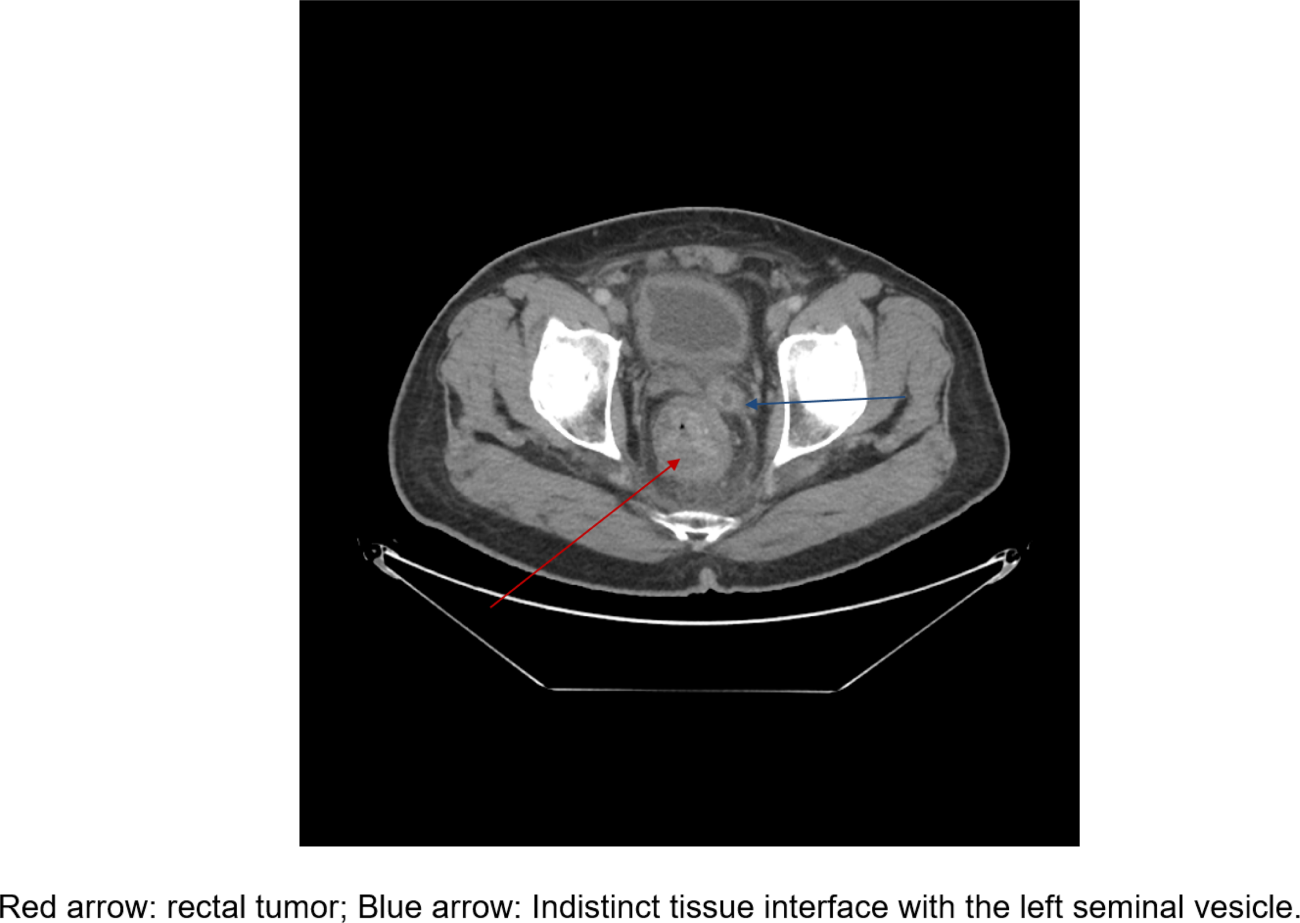
Preoperative CT showing diagnostic ambiguity. The red arrow marks the primary rectal cancer. The blue arrow points to an indistinct boundary between the rectum and the left seminal vesicle. This feature highlights a clinical diagnostic dilemma: the radiological challenge of definitively differentiating between malignant extramural invasion and a localized inflammatory or tuberculous collection prior to histological confirmation.

Over the subsequent three months (Months 0–3), a standard neoadjuvant approach was initiated to achieve local tumor control and potentially downstage the tumor. The specific regimen included pelvic radiotherapy (45 Gy in 25 fractions) with concurrent Capecitabine sensitization (825 mg/m² bid, 5 days per week), followed by two cycles of the CAPEOX regimen (Oxaliplatin 130 mg/m² on day 1; Capecitabine 1000 mg/m² bid on days 1–14, q3w). At Month 3, the patient developed severe grade 3 myelosuppression. This hematological toxicity was promptly managed with subcutaneous injections of recombinant human granulocyte colony-stimulating factor (rhG-CSF) 300 μg once daily and prophylactic intravenous ceftriaxone 2 g once daily. Following the resolution of myelosuppression, the patient declined further systemic therapy and strongly requested definitive surgical intervention. Follow-up chest and abdominal CT scans showed no significant changes in the rectal tumor (**Figure 1**) or the solitary pulmonary nodule (**Figure 2**). Additionally, the boundary between the rectal lesion and the left seminal vesicle appeared indistinct, raising concerns for extramural invasion or abscess formation (**Figure 3**). Multidisciplinary consultation recommended proceeding with surgical treatment. It is important to note that the decision to proceed directly to surgery at this stage, rather than attempting to adjust the neoadjuvant regimen or pursue further diagnostic workup for the suspected metastasis, was primarily driven by the patient’s strong personal request and refusal of further preoperative systemic therapy. Laparoscopic exploration revealed adhesions between the tumor and the left pelvic wall. Given the preoperative clinical impression of suspected pulmonary metastasis, the intraoperative finding of dense pelvic adhesions, and the patient’s explicit preferences, a laparoscopic-assisted palliative Hartmann procedure (resection with distal closure and proximal stoma formation) was performed. A small amount of necrotic tissue and purulent exudate was observed at the site of adhesion. The patient recovered well postoperatively and was discharged five days after surgery.

Postoperative pathology: Hematoxylin and eosin (HE) staining revealed that the cancer cells were arranged in irregular glandular patterns. The neoplastic cells were voluminous, possessing prominently enlarged round, oval, or pleomorphic nuclei. Mitotic figures were present, and the cells exhibited significant atypia. In localized areas, mucin accumulation within the glandular lumens formed mucin lakes. The cancerous tissue had penetrated through the serosal layer of the bowel wall (ypT4a). Proximal, distal, and circumferential resection margins (CRM) were all negative for carcinoma, achieving an R0 resection. There was no evidence of lymphovascular invasion, although perineural invasion was suggested. No metastasis was found in the harvested lymph nodes (0/24, ypN0). The overall morphological changes were consistent with a post-neoadjuvant chemoradiotherapy state, though a specific tumor regression grade (TRG) was not assigned. Notably, some lymph nodes exhibited granulomatous lesions accompanied by necrosis, including epithelioid cells and multinucleated giant cells, which are morphologically consistent with features suggestive of caseous necrosis. The detailed results of the immunohistochemical staining are summarized in **Table 1**. Special stains, including PAS, Gram stain, Acid-fast stain, Periodic Acid-Silver Methenamine stain, and Alcian Blue stain, were all negative. While the presence of granulomatous inflammation prompted consideration of various differential diagnoses (including fungal infections, sarcoid-like reactions to the malignancy, or treatment-related changes), the morphological presence of extensive caseous necrosis was highly suggestive of tuberculosis. To further evaluate this, quantitative PCR was performed directly on the formalin-fixed paraffin-embedded (FFPE) tissue from the resected surgical specimen, which returned positive for TB DNA, providing molecular evidence supporting tuberculosis. Taken together, these findings support the diagnosis of tuberculosis based on integrated histopathological and molecular evidence, although definitive microbiological confirmation was not available.

**Table 1:** Immunohistochemical staining results of the rectal specimen.

Marker	Result	Marker	Result
MLH1	Positive (+)	CgA	Negative (-)
MSH2	Positive (+)	CK8/18	Positive (+)
MSH6	Positive (+)	CDX-2	Mostly Positive (+)
PMS-2	Positive (+)	CD34	Positive (+) (Vascular)
C-erbB-2	1+	D2-40	Positive (+) (Lymphatic)
Ki67	Positive (+) 90%		
CD56	Focal Positive (+)		

Ten days after surgery (Postoperative day 10), and five days after removal of the perineal drainage tube, the patient noted pale yellow discharge from the anus. At that time, the perineal wound was healing well, and there was no notable pelvic or perineal discomfort. Pelvic CT showed only expected postoperative changes, without definite evidence of abscess formation, exudation, or other major abnormalities. Given the postoperative pathological findings of granulomatous inflammation with necrosis and positive TB DNA PCR in the resected specimen, the postoperative anal discharge was clinically suspected to represent a tuberculosis-related presacral infectious process rather than a radiologically confirmed abscess. We acknowledge that this diagnosis remained presumptive, because mycobacterial culture and acid-fast staining of the drainage fluid were not performed. To manage this suspected infectious complication, the presacral space was irrigated with 250 mL of normal saline daily for approximately 5 days until the drainage fluid became clear. Simultaneously, a standard 6-month systemic anti-tuberculosis regimen was initiated. This consisted of a 2-month intensive phase (Isoniazid 300 mg QD, Rifampin 600 mg QD, Pyrazinamide 1250 mg QD, and Ethambutol 750 mg QD), followed by a 4-month continuation phase (Isoniazid 300 mg QD and Rifampin 600 mg QD). The patient was closely monitored for liver function and medication tolerance throughout the treatment course. The patient demonstrated excellent adherence to the 6-month anti-tuberculosis regimen. During the treatment, mild liver function abnormalities were observed; however, these were effectively managed with oral bicyclol and magnesium isoglycyrrhizinate. The hepatotoxicity remained within an acceptable range, and the anti-tuberculosis therapy was completed without dose modification or interruption. Three months later (Postoperative month 3), follow-up evaluations revealed no increase in clinically suspected pulmonary metastasis or tumor recurrence. The patient subsequently underwent radiofrequency ablation (RFA) for the clinically suspected pulmonary metastatic lesion. Regarding long-term follow-up, the patient continued his postoperative care and 6-month anti-tuberculosis regimen at a local hospital due to geographical constraints. At the one-year follow-up, the patient reported a satisfactory quality of life. Owing to comprehensive preoperative education, he demonstrated good physiological and psychological tolerance to the stoma resulting from the Hartmann procedure. He had completed the anti-tuberculosis treatment and reported no recurrence of tumor or tuberculosis-related symptoms. The timeline of the patient’s clinical course, including diagnosis, treatment, and follow-up, is summarized in **Table 2**.

**Table 2:** Timeline of clinical events.

Timeline (Relative to initial presentation)	Clinical event	Intervention / observation
> 3 Months prior to Month 0	Symptom onset	Intermittent hematochezia and tenesmus.
Month 0	Initial presentation & Diagnosis	Clinical and imaging evaluation; diagnosed with locally advanced rectal adenocarcinoma (cT4bN2) and suspected pulmonary metastasis.
Month 0 to Month 1	Neoadjuvant Chemoradiotherapy	Pelvic radiotherapy (45 Gy/25f) with concurrent oral Capecitabine.
Month 1.5 to Month 2	Consolidation Chemotherapy	Received 1st and 2nd cycles of the CAPEOX regimen.
Month 3	Treatment Interruption	Developed severe grade 3 myelosuppression; chemotherapy halted. Re-staging scans showed stable disease.
Month 3.5 (Post-op Day 0)	Surgical Intervention	Underwent laparoscopic-assisted palliative Hartmann procedure.
Post-op Day 10	Postoperative Complication	Presentation of pale yellow anal discharge; a tuberculosis-related presacral infectious process was clinically suspected based on postoperative pathological findings and positive TB DNA PCR from the resected specimen, although pelvic CT did not show a definite abscess and no microbiological confirmation of the drainage fluid was obtained.
Post-op Day 10 to Day 15	Abscess Management	Daily saline irrigation of the presacral space until drainage cleared.
Post-op Day 10 onwards	Anti-TB Therapy	Initiation of a standard 6-month systemic anti-tuberculosis regimen.
Post-op Month 3	Follow-up & Intervention	No tumor recurrence or TB progression. Radiofrequency ablation (RFA) was performed for the clinically suspected pulmonary metastatic lesion.
1 Year Post-op	Long-term Follow-up	Favorable prognosis; no evidence of tumor recurrence or new tuberculous lesions.

## Patient perspective

During the telephone follow-up, the patient shared his perspective on the treatment process. He expressed that his overall subjective well-being was fair. Although he occasionally experiences mild fatigue, he has adapted well to the stoma and finds it acceptable in his daily life. Due to geographical constraints, the patient continues his recovery locally, and while formal quality-of-life scales were not administered, his verbal feedback indicates a positive attitude toward the clinical outcomes and the management of his concurrent conditions.

## Discussion

Rectal cancer coexisting with rectal and lymph node tuberculosis (TB) is a rare clinical entity that presents a profound diagnostic challenge. In our case, the preoperative colonoscopy confirmed adenocarcinoma but failed to establish the concurrent tuberculous component, as the scattered pale-red mucosal areas were visually misinterpreted as peritumoral inflammation. As highlighted in a recent review by Kudu et al. (6),gastrointestinal tuberculosis often presents with nonspecific symptoms and lacks a single, definitive diagnostic test, leading to a high risk of delayed diagnosis. In our patient, the overwhelming clinical and radiological features of locally advanced rectal cancer completely masked the occult tuberculous infection, illustrating the severe limitations of relying solely on standard oncological workups in such complex cases.

## Limited narrative review

To contextualize the management challenges of this case, we conducted a limited narrative review of the literature, focusing on cases of concurrent adenocarcinoma and histologically confirmed tuberculosis (literatures are summarized in **Table 3**). It should be noted that although some of the cited cases exhibit heterogeneity in their primary tumor pathologies (e.g., collision tumors or metastatic anal fistula cancer), they demonstrate highly consistent clinical patterns regarding diagnostic delays and complex treatment dilemmas. A prominent theme across several comparable reports is the distinct pattern of diagnostic delay. Notably, the diagnosis of tuberculosis is frequently not established until the intraoperative or postoperative phase. For instance, Tokuno et al. (8) reported a case of ascending colon cancer where preoperative biopsies confirmed adenocarcinoma, yet the coexisting intestinal tuberculosis was only discovered in the final pathological examination of the resected specimen. Similarly, Lin et al. (9) described a more heterogeneous sigmoid “collision tumor” case involving lymphoma and adenocarcinoma components; although the tumor pathology was highly complex, the concurrent tuberculosis infection similarly evaded routine preoperative workups and was only definitively identified via PCR on the resected specimen, further illustrating the occult nature of low-grade TB in a malignant background. These findings strongly support our observation that standard preoperative workups for rectal cancer often fail to detect concurrent low-grade tuberculous infections.

**Table 3:** Summary of case reports on colorectal cancer complicated by tuberculosis.

Author / year	Patient (age/sex)	Primary clinical manifestations	Timing of TB diagnosis	Imaging / endoscopic features	Treatment method	Outcome
Liu et al. (2022) (10)	71 / F	Intermittent hematochezia (1 year).	Intraoperative / Postoperative (Discovered nodules during surgery; confirmed by biopsy)	Pre-op dx: Low rectal cancer. Intra-op: Diffuse nodules mimicking metastasis.	Anti-TB therapy (primary), followed by Abdominoperineal Resection (secondary).	Discharged in good condition; surgery successful after TB control.
Ahmad et al. (2024) (14)	49 / M	Constipation, abdominal pain.	Preoperative (1st biopsy: TB; 2nd biopsy: Adenocarcinoma)	CT: Annular thickening of ascending colon with infiltrates.	Right hemicolectomy + Post-op anti-TB therapy. (No chemotherapy).	Good early prognosis; TB and cancer detected early.
Tokuno et al. (2025) (8)	67 / M	Positive fecal occult blood.	Postoperative (Pre-op biopsy: Adenocarcinoma; Post-op path: Cancer + TB)	Colonoscopy: 2 circular narrowing sections and ulcers.	Laparoscopic right colectomy.	Diagnosed as ascending colon cancer with intestinal TB.
Aibara et al. (2017) (12)	87 / F	Early rectal cancer with massive ascites.	Preoperative (Via Staging Laparoscopy biopsy)	PET-CT: High uptake in hypertrophic peritoneum (suspicious for TB peritonitis).	Anti-TB therapy (4 months) →Laparoscopic Low Anterior Resection.	Successful treatment; laparoscopy assessed TB status before cancer surgery.
Lin et al. (2014) (9)	81 / M	Abdominal pain (Sigmoid collision tumor).	Postoperative (PCR of resected colon specimen)	Not detailed (Collision tumor of Lymphoma + Adenocarcinoma).	Surgery + Systemic Chemotherapy + Anti-TB therapy.	Died (Advanced colon cancer with lung metastasis).
Murata et al. (2014) (13)	69 / M	Perianal pain, anal fistula.	Preoperative (CT confirmed Pulmonary TB; MRI confirmed Rectal tumor)	MRI: Anal fistula + Rectal tumor. CT: Pulmonary TB.	Seton drainage → Anti-TB (2 mo) → Rectal resection → Chemo.	No recurrence at 6 months; tumor shrunk after neoadjuvant therapy.

Beyond delayed identification, the visual mimicry of tuberculosis can lead to intraoperative staging errors that drastically alter surgical management. Liu et al. (10) described a patient diagnosed with low rectal cancer where diffusely distributed nodules found during laparoscopic exploration were initially mistaken for extensive peritoneal metastasis. This visual resemblance to Stage IV carcinomatosis led to the abandonment of radical surgery in favor of biopsy, which subsequently revealed tuberculous granulomas. This case illustrates that concurrent tuberculosis does not merely coexist but can actively mimic advanced malignancy, complicating intraoperative decision-making. Furthermore, preoperative imaging and endoscopy often fail to differentiate the two conditions due to overlapping features like wall thickening and lymphadenopathy. While Gharbi et al. (11) noted that some TB lesions might extrude caseous fluid upon biopsy, such specific signs are rarely observable when a malignant mass dominates the endoscopic field.

The timing of the TB diagnosis fundamentally alters the treatment trajectory, creating a divergence in management strategies. When tuberculosis is suspected preoperatively, a “TB-first” strategy is often advocated to reduce surgical risks. Aibara et al. (12) successfully utilized staging laparoscopy to diagnose tuberculous peritonitis in a rectal cancer patient, allowing for a four-month course of anti-tuberculosis therapy prior to performing a curative laparoscopic low-anterior resection. Similarly, Murata et al. (13) managed a patient with rectal adenocarcinoma, an anal fistula (later confirmed as metastatic anal fistula cancer), and concurrent pulmonary tuberculosis, by administering anti-tuberculosis therapy for two months before surgery, aiming to minimize infection-related complications. Conversely, when the diagnosis is delayed until after surgery, as in our case, the lack of preoperative anti-tuberculosis coverage can lead to severe postoperative infectious complications. In our patient, this likely contributed to the development of a postoperative presacral abscess. Furthermore, even if the concurrent tuberculosis is successfully diagnosed preoperatively, it can still create profound treatment dilemmas. Ahmad et al. (14) reported a case of concurrent colon adenocarcinoma and tuberculosis where despite early detection, adjuvant chemotherapy was withheld entirely due to concerns regarding drug interactions with the necessary anti-tuberculosis regimen. Specifically, it is plausible that the neoadjuvant CAPEOX chemotherapy and concurrent pelvic radiotherapy contributed to the reactivation of latent tuberculosis (15). Cytotoxic chemotherapy and radiation induce profound systemic immunosuppression (16)—as clearly evidenced by the patient experiencing severe grade 3 myelosuppression in this case. This immunosuppressive state severely impairs T-cell-mediated immunity, which is crucial for maintaining dormant mycobacteria within granulomas. Consequently, the breakdown of these immune barriers, compounded by the physiological stress and local tissue disruption from the surgical procedure, likely facilitated the reactivation of latent TB within the pelvic lymph nodes, culminating in the formation of the presacral abscess. This highlights the critical necessity of considering TB reactivation in the differential diagnosis of postoperative complications, especially in patients with a prior history of pulmonary infection who undergo immunosuppressive neoadjuvant therapies.

This study has several limitations. First, as a single case report, the findings should be interpreted as clinically suggestive rather than generalizable evidence. Although the case is relevant to oncological practice in tuberculosis-endemic settings, it cannot establish the frequency, risk factors, or optimal management strategy for rectal cancer complicated by concurrent tuberculosis. Second, the diagnosis of tuberculosis was based on integrated histopathological and molecular evidence, including granulomatous inflammation with necrosis and positive TB DNA PCR from the resected FFPE specimen. However, acid-fast staining was negative, and mycobacterial culture, interferon-gamma release assay, preoperative sputum testing, and formal infectious disease consultation were not performed because tuberculosis was not suspected before surgery. Therefore, the microbiological profile of the infection could not be fully characterized, and other granulomatous conditions, although considered less likely, could not be completely excluded. Third, the postoperative anal discharge and suspected presacral infectious process were clinically attributed to tuberculosis on the basis of the resected-specimen findings and the response to local irrigation plus systemic anti-tuberculosis therapy; however, no microbiological confirmation of the drainage fluid was obtained, and a definite abscess was not radiologically confirmed. This limits the certainty with which the postoperative complication can be classified as a tuberculous presacral abscess. Fourth, the available histopathological images mainly demonstrated the adenocarcinoma component, whereas high-quality images directly showing granulomatous inflammation or caseous necrosis were limited. Finally, although the patient developed grade 3 myelosuppression during neoadjuvant chemoradiotherapy, this report cannot determine whether occult tuberculosis or its associated inflammatory burden contributed to hematological intolerance. The possible interaction between latent tuberculosis, cancer treatment-related immunosuppression, and postoperative infectious complications requires further investigation in larger studies.

Rectal cancer concurrent with rectal and lymph node tuberculosis is a rare but critical clinical entity. The dominance of malignant features often masks the infectious pathology, leading to missed preoperative diagnoses and increased postoperative morbidity. Therefore, the primary takeaway from this case is that tuberculosis should remain in the differential diagnosis when rectal cancer is accompanied by atypical mucosal findings, granulomatous pathology, discordant staging features, a previous tuberculosis history, or postoperative infectious complications that are not readily explained.

## Data Availability

The original contributions presented in the study are included in the article/supplementary material. Further inquiries can be directed to the corresponding author.
